# From Isotropic to Anisotropic Side Chain Representations: Comparison of Three Models for Residue Contact Estimation

**DOI:** 10.1371/journal.pone.0019238

**Published:** 2011-04-28

**Authors:** Weitao Sun, Jing He

**Affiliations:** 1 Zhou Pei-Yuan Center for Applied Mathematics, Tsinghua University, Beijing, China; 2 Department of Computer Science, Old Dominion University, Norfolk, Virginia, United States of America; German Cancer Research Center, Germany

## Abstract

The criterion to determine residue contact is a fundamental problem in deriving knowledge-based mean-force potential energy calculations for protein structures. A frequently used criterion is to require the side chain center-to-center distance or the 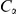-to-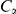 atom distance to be within a pre-determined cutoff distance. However, the spatially anisotropic nature of the side chain determines that it is challenging to identify the contact pairs. This study compares three side chain contact models: the Atom Distance criteria (ADC) model, the Isotropic Sphere Side chain (ISS) model and the Anisotropic Ellipsoid Side chain (AES) model using 424 high resolution protein structures in the Protein Data Bank. The results indicate that the ADC model is the most accurate and ISS is the worst. The AES model eliminates about 95% of the incorrectly counted contact-pairs in the ISS model. Algorithm analysis shows that AES model is the most computational intensive while ADC model has moderate computational cost. We derived a dataset of the mis-estimated contact pairs by AES model. The most misjudged pairs are Arg-Glu, Arg-Asp and Arg-Tyr. Such a dataset can be useful for developing the improved AES model by incorporating the pair-specific information for the cutoff distance.

## Introduction

The accurate identification of inter-residue contact is a crucial step in the understanding of protein structure. The residue contacts observed in crystal structures of globular proteins are generally considered the intrinsic inter-residue interactions. Based on this commonly accepted assumption, structures from the Protein Data Bank (PDB) [1] have been used to elucidate two-body residue contact and packing potentials since 1970s [2]. Miyazawa and Jernigan developed the theory of effective inter-residue energy from protein crystal structures [3], [4] based on the Bethe Approximation [5], [6], [7], [8] and quasi-chemical approximation [9], [10], [11], [12], [13]. Applying Boltzmann's law, Sippl proposed an approach to yield mean force potential of residue interactions as a function of distance [14]. In addition to the residue-distance-dependence studies [15], [16], the effect of relative orientations on contact energy has been investigated [17], [18]. In order to include the influence of multi-residue interactions and local environmental dependence, development of tri-residue [19], [20], [21], four-residue [22], [23], [24], [25], [26], [27], [28], [29], [30] and secondary structure-related energy [31] have been the focus of recent research.

For the sake of simplicity and computational efficiency, mean force potential are widely used in various applications, such as assessment of protein structures [32], [33], [34], [35], [36], folding recognition and threading [37], [38], [39], [40], [41], [42], [43], detection of native protein conformation [44], [45], [46], [47], [48], native topologies [49], [50], [51] and protein structure prediction [52], [53], [54], [55]. Mean force potentials use reduced representations for side chains.

The contact models can be classified into two broad categories: the all atom model and the reduced representation model. In the all atom model, a pairs of residues are considered in contact if any two non-hydrogen side chain atoms (NHSA) from residues 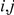 are within a specified cutoff distance [56], [57], [58], [59]. This model is expected to have accurate determination of the contact pairs [15], [47], [60], [61]. The drawback is that it requires the knowledge of location of all the atoms on the side chains, and that is computationally expensive in structure prediction. Popular reduced representation of the side chains have been proposed through the use of 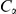 atom [62], [63], 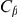 atoms, the centroid of amino acid and the centroid of side chain. Models with all atom main chain backbone and a single united atom for side chain have been proposed [53]. Another advanced model has been proposed with hydrogen bonds and flexible ellipsoidal side chains [64], [65], [66]. However, a more accurate description is required to capture atom-atom interactions in detail.

Two residue side chains are considered to be in contact if the side chain center or the 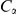 atom distance is less than a specified, pre-determined threshold distance [2], [3], [4], [14], [25]. The influence of a residue over surrounding medium can be effectively characterized at a limit distance [67], [68], [69], [70]. A cutoff distance of 8.0 Å has been used in multi-body potentials [27], folding rate of proteins [71] and protein stability [72], [73]. Other various contacting distances have also been used for protein-folding studies. A commonly used side chain center distance threshold is 6.5 Å [3], [4], [18]. Spatial contact is considered to exist if 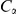 atom pair or 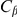 atom pair distance is less than 7.0 Å [74], [75], [76], [77].

In order to avoid the drawbacks of arbitrary cutoff distance, Yang, et. al. proposed the parameter-free elastic network model (pfENM) to improve the estimation of B- factor [78]. Although the artificial cutoff distance are not as perfect as we expected, these convenient criteria still find their applications in many fields, especially in protein structure networks [79], [80], [81]. Cutoff distance is crucial to the contact degree distribution function describing the network behavior.

It is challenging to find a cutoff distance due to the variation in sizes, the preferred orientations and the anisotropy nature of the side chains. However, it can be more and more accurate as the mechanism of the contact is more and more understood. This study compares the following three models: the surface-to-surface model based on the side chain Atom Distance Criteria (ADC), the Isotropic Sphere Side chain (ISS) model and the Anisotropic Ellipsoid Side chain (AES) model using a dataset of 424 high resolution proteins from the PDB. We derive a dataset and illustrate the pairs that were wrongly estimated using the AES model for future study to improve the AES model.

## Results and Discussion

It is known that side chains tend to have preferred orientations and exist as certain energetically favorable rotamers [82], [83], [84], [85], [86], [87], [88], [89]. This anisotropic nature of the side chains proposes challenges in the determination of the contact. In general, there are side chain overlaps (as defined by van der Waals radii) in experimentally determined NMR and crystallographic protein structures [90], [91]. But the number of steric clashes is low. Side chain overlaps defined by covalent radii are even less. We investigated the overlaps in the high resolution PDB structures using three side chain models. The dataset was used in our previous work [49] and it includes 424 protein structures with single-chain, higher than 1.5 Å in resolution, less than 30% sequence identity structures from the PDB that are determined using X-ray crystallography technique [1]. Some PDBs with missed NHSAs are excluded.

### Residue-contact distribution for the ADC model

In the ADC model, two amino acids overlap if any pair of atoms, one from each amino acid, is within the overlap cutoff distance. Two non-overlapping amino acids are in contact if any pair of atoms, one from each amino acid, is within the contact distance.

We calculated the overall residue contact degree 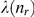 and the overlap degree 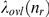 for 424 high-resolution PDBs based on ADC model. 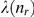 denotes the total number of contacts among all *n_r_* residues in a protein. The overlap degree 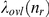 means the total number of side chain overlaps for a protein with *n_r_* residues. Since it is not expected to find large number of residue overlaps in the test dataset, the lower the 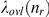, the more accurate contact model. The total number of residues falling within the contact distance of residue 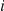 is recorded as the contact degree 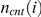.
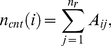
(1)

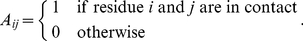
(2)Here 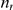 is the total number of residues in the protein. Residue 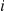 and its surrounding neighbors form a residue-contact cluster. This cluster is related to residue 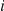 and contains 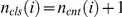 residues, in which the residue immediately before and after 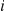 on the protein sequence are excluded.

**Figure 1:**
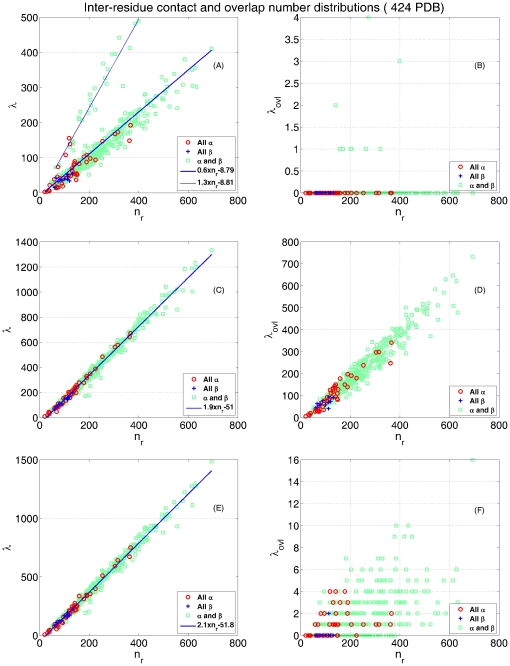
Residue contact distribution by ADC/ISS/AES contact model. 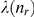
 and 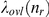 denotes the total number of contact and overlap among all residues in the protein. Surface gap distance 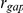 = 0.0 Å is used here. Data points for all-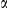 helix, all-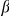 sheet, 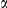 helix-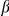 sheet proteins are plotted in different marker styles. (A)(B) ADC model; (C)(D) ISS model; (E)(F) AES model.

An overall residue contact degree 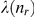 can be described by the size of the contact network.
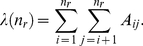
(3)

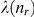
 provides an intuitive understanding of the compactness and overall connectivity. In the same way, overlap degree 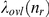 can be defined to describe the side chain overlaps.
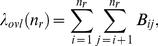
(4)

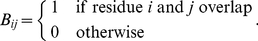
(5)The ADC model reveals a linear relation between the contact degree 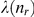 and the protein length 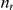 (**Figure 1** (A)). Linear fitting formula 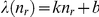 reveals a spontaneous collapse character of protein structures in different sizes. If all data points are matched simultaneously, the linear relationship can be described by 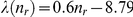 (the lower matching line in **Figure 1** (A)) with a confidence 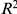 = 0.73. 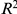 is the fitting coefficient of determination. The data fitting is facilitated by the Matlab fit function [92]. The relatively low fitting confidence is the result of the deviation of some ‘escaping’ data points.

An interesting observation is that the ADC model has the ability to classify protein structures. The ‘escaping’ data points, depicted in **Figure 1** (A), constitute another group and have a distinct linear slope. Thus, 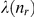 is divided into two separate groups with obviously different slopes. Here we use the linear slope 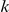 as a criterion. To separate the data into two groups, we used the line that fit all the data points as a reference, where 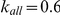 and 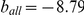. The slope of the data point 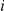 is calculated as 
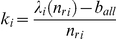
. If 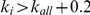, the data point 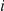 is placed in another group. When all the ‘escaping’ data points are fitted as a separate group, the linear regression is 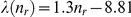 with a confidence 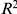 = 0.93 (the upper matching line in **Figure 1** (A)).

Detailed evaluation suggests that proteins with steeper increasing slopes are highly compact and can be considered dense-core proteins [93]. These well-packed structures can roughly be classified into three categories: (1) nearly-perfect globular proteins with short and flexible secondary structures; (2) proteins composed of a bundle of tightly packed alpha helixes; (3) proteins composed of curly 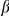 sheets.

**Figure 1** (B) shows the distribution of the overlaps in the test data set. The fact that very few proteins have overlaps in the dataset suggests that ADC is an accurate side chain model that can be used to reflect the anisotropic effect of the residue side chains. In fact, the largest number of overlaps is 4 in one protein among the entire dataset. In addition, the sparse and random data distributions suggest that systematic misinterpretation of side chain overlaps is avoided in the ADC model.

The residue contact number depends on the 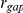, which is included in the definition of residue contact cutoff distance 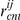 (Equation (9)). Here 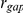 is a gap distance between atom surfaces in ADC criteria (see Methods section). In order to investigate the influence of 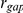 on overall residue-contact distributions, 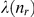 and 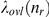 are calculated for 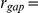
0.5 Å, 1.0 Å, 1.5 Å and 2.0 Å, respectively. The contact cutoff distance 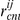 increases as the 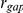 increases. Residue pairs with larger distance, which were considered as separated-residues pair, are included as contact pairs. Not all these extra contact pairs reflect intrinsic residue interactions. An appropriate 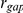 value is required to eliminate unexpected contacts. In the ADC fixed length model section, the derivation of the optimal 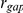 is presented. Since the overlap cutoff distance is independent of the 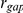, the number of residue overlaps remain unchanged as the 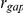 increases.

### Residue-contact distribution for the ISS model

**Figure 2:**
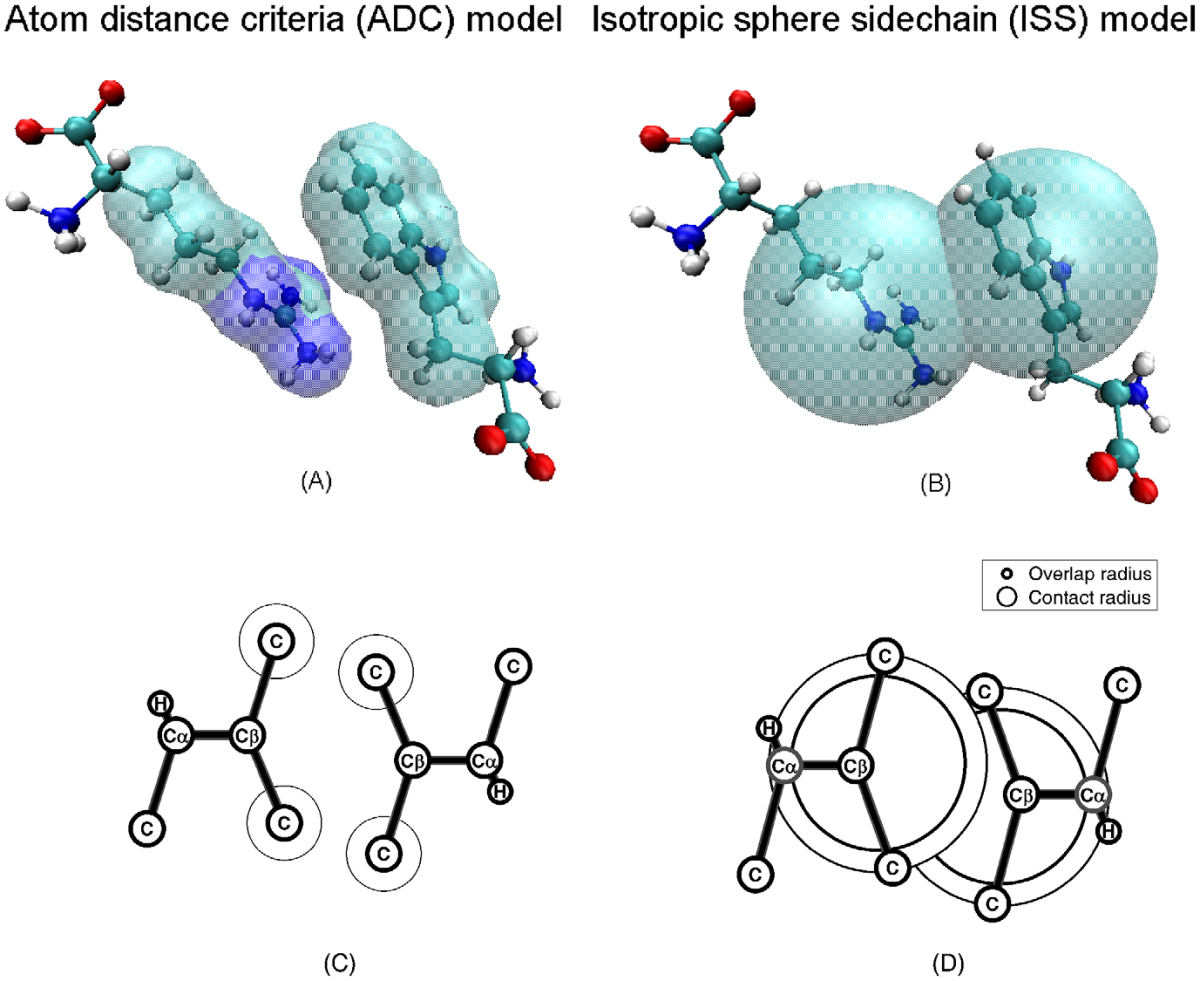
Residue side chain contact model. (A) All-atom sidechain model; (B) Simple isotropic sphere side chain model will cause spurious overlaps; (C) Effective overlap radius and contact radius of residue side chain atoms in the ADC contact model. (D) Effective overlap radius and contact radius in the ISS model.

The ISS model uses a sphere to represent the side chain. This simple model can result in spurious side chain overlap as shown in **Figure 2**(A)–(B).

In this study, we used the geometry center 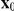 of heavy-atoms as the center of side chain.
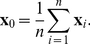
(6)Here 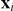 is the coordinate of atom 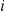. 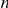 is the number of heavy atoms.

In the ISS method, the error caused by neglecting side chain anisotropy can also be observed in the distributions of 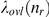, which increases linearly with respect to protein size (**Figure 1**(D)).

It is not surprising that the bulky side chains, such as Trp and Arg, lead to more significantly spurious overlaps. For example, phenyl rings (**Figure 2**(A)) prefer to form parallel or vertical orientations, and the spherical representation can overestimate the size of it. We also observed that the two lines fitted in the contact degree (Figure 1(A)) appear as one line (Figure 1(C)) in a linear regression of 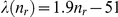 with a confidence 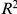 = 0.99. The sensitivity to anisotropy and ability to discriminate among different structure packings are lost in the ISS model. The 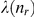 behavior of the ISS model with a surface-gap distance 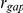 = 0.0 Å appears to be equivalent to that in the ADC model with an atom surface-gap distance of 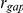 = 1.0 Å or 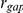 = 1.5 Å.

We also calculated the distributions of 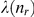 and 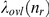 with 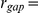 0.5, 1.0, 1.5 and 2.0 Å respectively for the ISS model (data not shown). The increase in 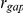 leads to a simultaneous increase in the residue-contact cutoff distance 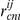. As a consequence, more residue side chain pairs are considered to be within the contact range. However, 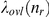 distributions do not change with different 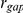 for the reason that the overlap cutoff distance 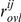 is independent of 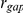 (see Equation (15)). For all types of 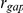, ISS model has significantly more overlaps than ADC model.

### Residue-contact distribution for the AES model

In AES model, the residue side chain is represented as an ellipsoid with anisotropic radii in three principal dimensions. An ellipsoid collision-detection algorithm [94], [95] was used to determine the side chain contact and overlap. With the anisotropic radii in three principal dimensions, the AES model is much more accurate than the ISS model. Although the AES still has false positive determination of overlaps, the number of misjudgements is less than 5% of that in the ISS model.

**Figure 1**(E)–(F) show the distribution of 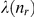 and 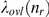 calculated by the AES model. The 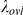
−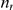 ratio for the AES (**Figure 1**(F)) is much less than that in the ISS model (**Figure 1**(D)). With the ellipsoid overlap criterion, more than 95% of the false residue overlaps in the ISS model have been avoided, and most of the 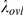 are less than 10. This number appear to be less than that in previous works [96].

Although the side chain anisotropy is taken into consideration to some extent, the 20 types of side chain conformations are still encompassed in a quadratic surface. The bulky side chain volume will lead to an underestimation of residue side chain distances. As a result, many closely packed residue pairs are mistakenly judged as overlaps. The anisotropic ellipsoidal radii help to improve accuracy in discriminating contact-residue pairs from separate-residue pairs in the AES model. However, the AES model still encounters difficulties in assessing the difference between overlap and contact. Part of residue contacts are taken as overlaps.

We analyzed contact determination algorithms with the three models (see method section). The ISS model is the least computationally intensive, followed by ADC and then AES. The accuracy rank is ADC, AES, ISS from high to low respectively. Algorithm accuracy/computing-cost ratio suggests that the ADC model is a cost effective model with the best accuracy. AES model is the most computational method among the three because the detection of ellipsoid collision algorithm is the most intensive step and needs to be improved in the future.

**Figure 3:**
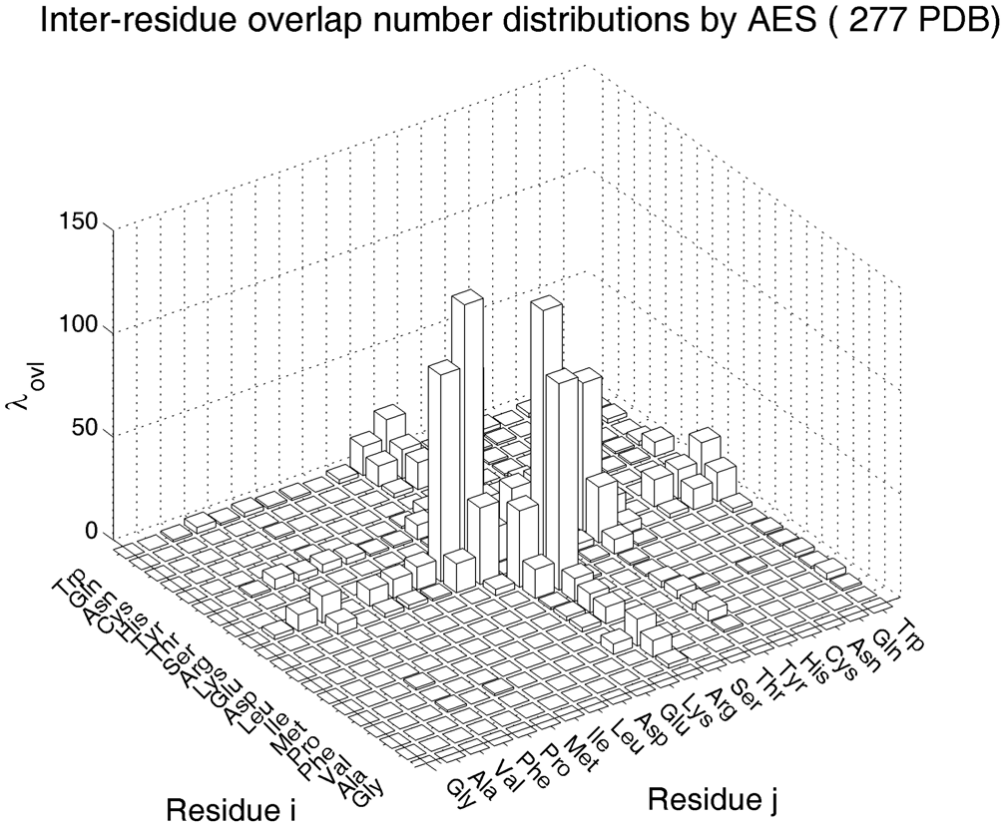
Overlap distributions of 20×20 residue pairs for AES model. AES-determined overlaps emerge in 277 out of 424 PDBs.

**Figure 4:**
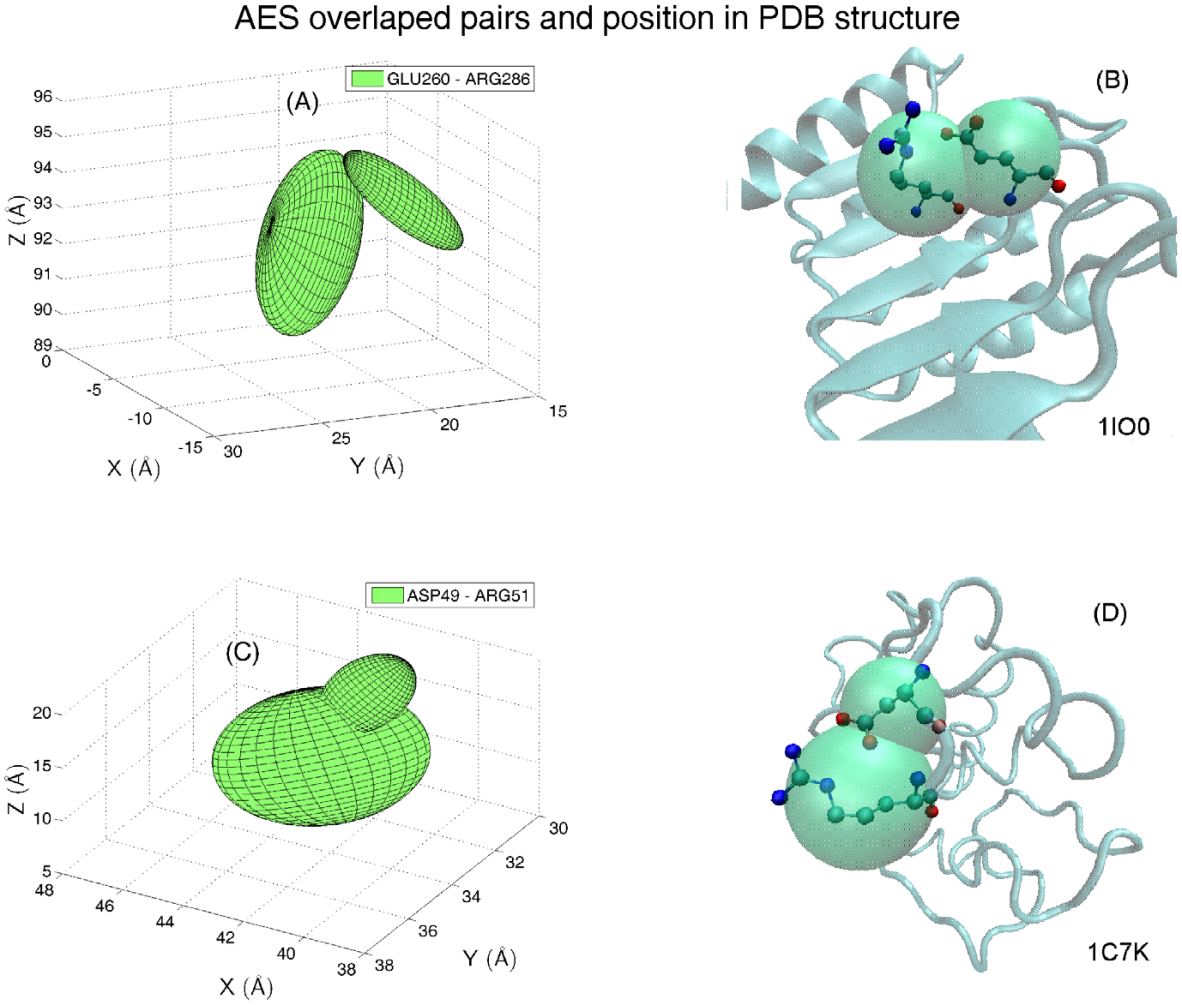
Examples of the ellipsoid overlap and side chain positions in PDB structures. (A) Glu-Arg overlap in 1IO0; (B) Glu260-Arg286 side chain postions in 1IO0 ; (C) Asp-Arg overlap in 1C7K; (D) Asp49-Arg51 side chain postions in 1C7K.

An analysis of the number of overlaps determined using the three models show that less than 50% of the total pairs of ADC contact are correctly predicted by ISS model. Whereas most of the 424 proteins have more than 95% ADC contacts shared by AES model. **Figure 3** shows the overlap number distribution for the 20×20 pairs of residues using the AES model. It appears that AES model is successful in determination of contact for most of the pairs. However, AES fails in most of the pairs involving Arg-Glu, Arg-Asp and Arg-Tyr. Arg-Glu pair is one of the most frequently seen false positive overlaps due to their large side chains. **Figure 4** (A) illustrated the ellipsoids calculated for an Glu-Arg pair. It appears that the overlapping volume is not much in this case. In another example of Asp-Arg (**Figure 4**(C)), the false positive overlap involves quite a lot of overlapping volume. **Figure 4**(B)(D) show the all-atom side chain positions of residue pair Glu260-Arg286 in 1IO0 (PDB ID) and residue pair Asp49-Arg51 in 1C7K (PDB ID). It is possible to develop an improved AES model that involves pair-specific and relative orientation dependent distance criteria for more accurate representation of the side chains.

### Pair-specific contact cutoff distance

A popular contact cutoff is 5 Å between two NHSA atoms. We investigate if this threshold is a good estimation for all pairs of residues in this section. In theory, the cutoff distance in the ADC model should depend on the specific radii of atoms that are in contact and the atom surface gap distance 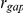. This is because the two residues can interact through different pairs of atoms. For example, the minimal distance of Val–Phe may occur either between atom pairs CG1–CE1 or CG2–CZ. The contact/overlap distances are distributions, rather than a single value (such as 5 Å). The relation between 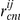 distributions and the 5 Å model is an interesting topic. In addition, the method of how to estimate an appropriate 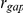 value for the cutoff distance is discussed in this section.

The interactions between two residues have preferred distances and orientations, rather than a random packing. For any two residues, the most frequently occurring residue distance is considered its major contact distance. As the two residues depart from, or come close to, each other, the occurrence probability decreases gradually and the interaction energy becomes relatively unstable till the contact distance increases to the upper limit, the cutoff distance.

**Figure 5:**
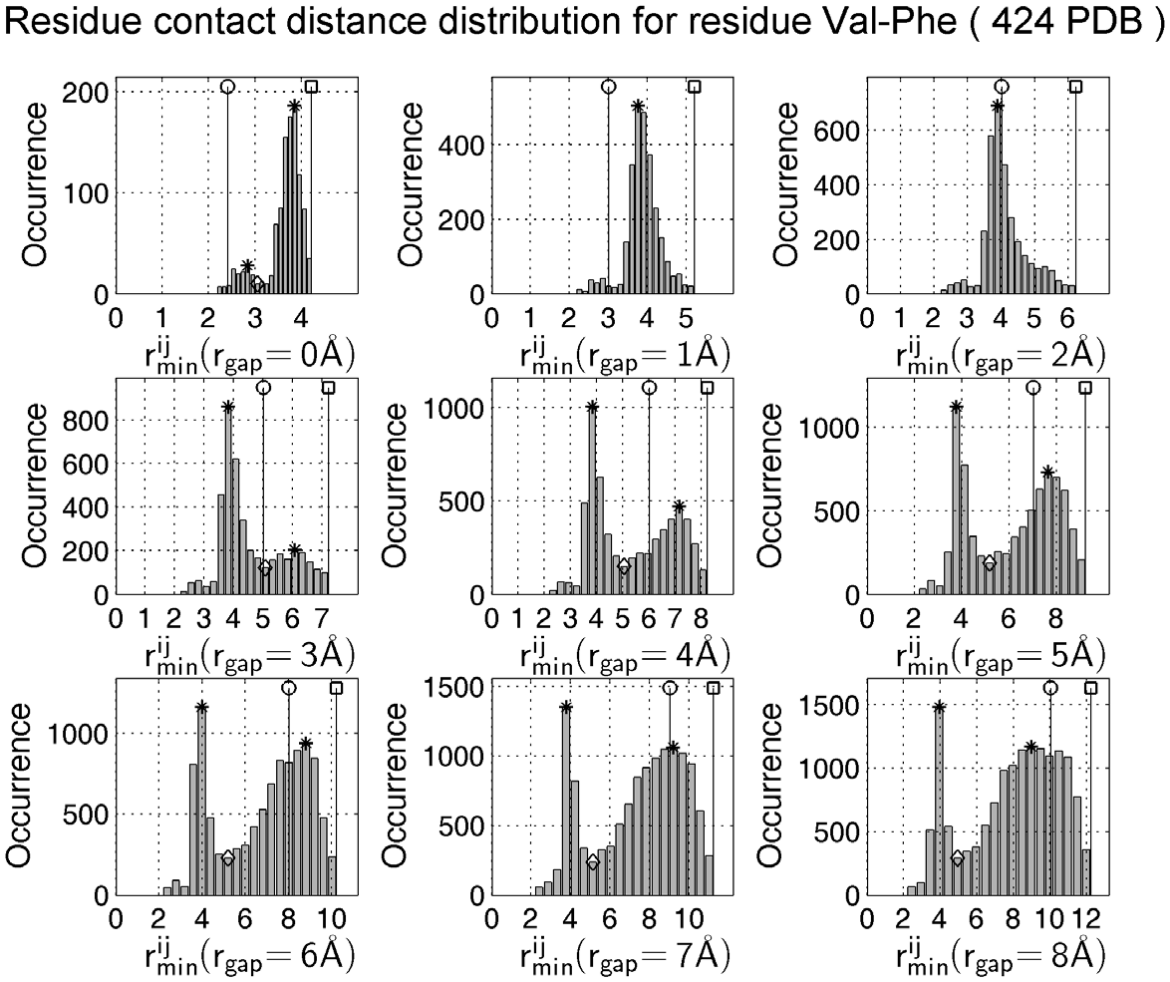
The contact distance distribution for residue pair Val–Phe at difference gap distances. The stem with circle indicates the minimal cutoff distance 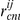. The stem with a square indicates the maximal cutoff distance. The stars indicate high occurrence peaks. The diamonds indicate occurrence valley position. The 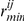 is the minimal atom-to-atom distance between Val and Phe side chains. As the gap distance 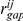 increases from 0 to 8 Å, the cutoff distance 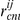 increases simultaneously.

**Figure 6:**
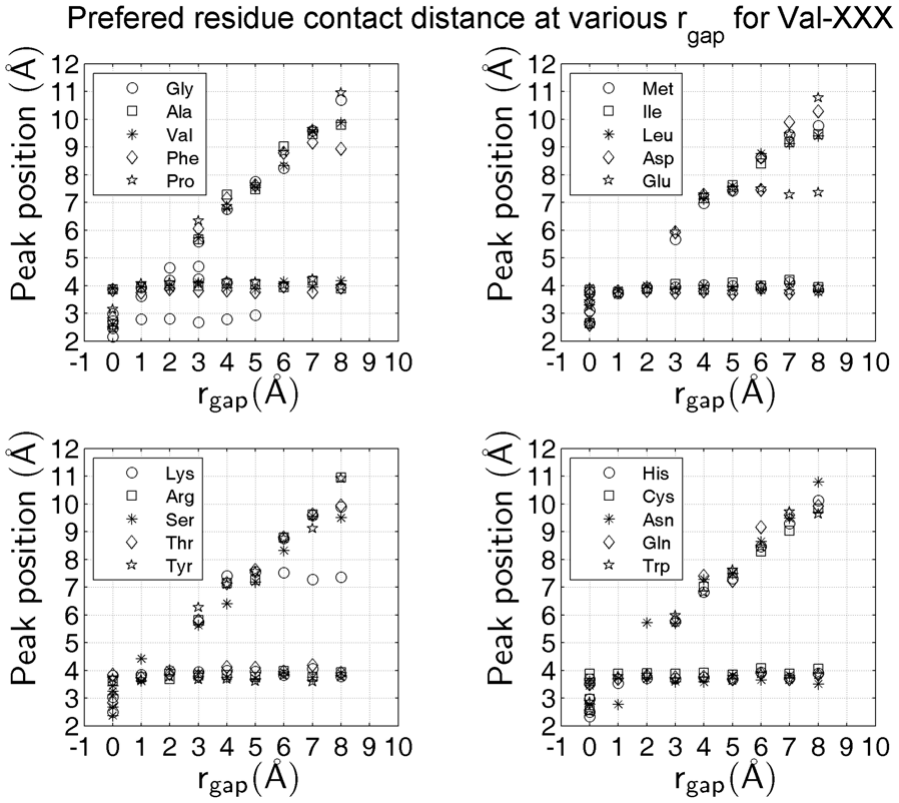
The preferred contact distance for Val-involved residue pairs at different gap distances. The positions of occurrence peaks in Val–XXX contact-distance distribution show two different behaviors. One is the stable, high occurrence-peak positions (the preferred contact distances) close to 4 Å, which are independent of the gap distance 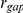. The other is the linearly increasing peak positions (contact distances caused by increase in 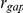).

**Figure 5** depicts the contact-distance distribution of Val-Phe pair. The histogram of packing distances between Val and Phe is a Gaussian-shaped function with a peak close to 3.9 Å. The peak position, i.e. the preferred contact distance, is almost independent of the cutoff distance. Only when the gap distance 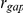 is beyond 3 Å will a second peak arise gradually. From the linearly increasing manner and peak-position shift, we ascertained that this peak (‘non-local contact’) is the result of the increase of cutoff distance, rather than an intrinsic interaction between Val and Phe. We further investigated all the pairs involving Val. The peak distributions of all residue pairs containing Val confirm the steady behavior of residue contact (**Figure 6**). As the gap distance 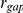 increases, the peak positions corresponding to the preferred Val–XXX contact distance remain constant. While the positions of ‘non-local contact’ peak increase linearly with respect to 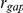.

The peak distributions allow us to set the cutoff distance for residue contacts. Between the two contact peaks, there is a low occurrence valley close to 5 Å (**Figure 5**). The valley position provides a rough estimation of the cutoff distance. We determined the cutoff distance for all the 210 pairs of residues using the position of the valleys (Table 1). The popular cutoff distance of 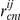 = 5 Å appears to be effective in most of the pairs. However, some residue pairs such as Gly-XXX have complicated distributions with multiple valleys. In such cases, a larger cutoff distance will be chosen as the optimal value such that all the preferred contact distances (the stable peaks) can be included.

**Table 1:** The optimal atom–atom cutoff distance for all types of residue side chain contact pairs (Unit: Angstrom).

ID	Gly	Ala	Val	Phe	Pro	Met	Ile	Leu	Asp	Glu	Lys	Arg	Ser	Thr	Tyr	His	Cys	Asn	Gln	Trp
**Gly**	3.9	5.8	4.4	3.7	5.4	4.8	6.2	4.2	4.6	5.5	4.6	5.0	4.1	5.8	5.5	3.9	5.6	4.1	4.5	3.5
**Ala**	5.8	5.1	5.0	5.1	4.7	6.0	5.1	4.9	5.0	4.9	5.0	4.5	4.9	5.0	4.8	4.8	5.0	4.8	5.2	5.0
**Val**	4.4	5.0	5.2	5.1	5.2	5.0	5.1	5.2	4.7	5.0	5.0	4.8	5.0	5.1	5.2	5.1	5.1	4.9	5.0	4.9
**Phe**	3.7	5.1	5.1	4.9	4.8	5.1	5.2	5.2	4.9	4.6	4.8	5.1	4.9	4.9	4.9	4.6	4.7	5.0	4.9	5.4
**Pro**	5.4	4.7	5.2	4.8	4.8	5.0	5.0	5.1	4.6	5.1	4.8	4.8	4.7	5.1	4.8	5.2	4.9	4.5	4.7	5.1
**Met**	4.8	6.0	5.0	5.1	5.0	5.7	5.4	5.2	4.5	4.5	5.5	4.7	4.9	4.9	5.4	5.7	5.0	4.7	5.1	4.9
**Ile**	6.2	5.1	5.1	5.2	5.0	5.4	5.2	5.5	4.7	4.8	5.0	5.1	4.8	5.1	5.2	4.7	5.4	4.9	5.3	4.9
**Leu**	4.2	4.9	5.2	5.2	5.1	5.2	5.5	5.5	4.5	5.0	5.0	5.0	4.8	4.9	5.2	5.1	4.9	5.0	4.8	5.4
**Asp**	4.6	5.0	4.7	4.9	4.6	4.5	4.7	4.5	4.9	4.5	4.4	4.5	5.0	4.5	4.8	4.5	4.4	4.5	4.9	4.7
**Glu**	5.5	4.9	5.0	4.6	5.1	4.5	4.8	5.0	4.5	5.0	5.0	4.9	5.1	4.8	4.7	5.0	4.4	4.3	4.6	5.1
**Lys**	4.6	5.0	5.0	4.8	4.8	5.5	5.0	5.0	4.4	5.0	4.8	4.7	4.4	4.9	4.8	4.8	5.0	4.4	4.5	5.0
**Arg**	5.0	4.5	4.8	5.1	4.8	4.7	5.1	5.0	4.5	4.9	4.7	5.0	4.5	4.7	4.7	4.5	5.0	4.9	4.9	4.6
**Ser**	4.1	4.9	5.0	4.9	4.7	4.9	4.8	4.8	5.0	5.1	4.4	4.5	4.6	4.5	4.7	4.6	5.0	4.4	5.1	5.1
**Thr**	5.8	5.0	5.1	4.9	5.1	4.9	5.1	4.9	4.5	4.8	4.9	4.7	4.5	5.0	4.7	4.6	5.0	4.7	4.7	4.7
**Tyr**	5.5	4.8	5.2	4.9	4.8	5.4	5.2	5.2	4.8	4.7	4.8	4.7	4.7	4.7	5.2	4.7	5.4	4.6	4.7	5.3
**His**	3.9	4.8	5.1	4.6	5.2	5.7	4.7	5.1	4.5	5.0	4.8	4.5	4.6	4.6	4.7	4.6	5.1	5.2	4.8	4.9
**Cys**	5.6	5.0	5.1	4.7	4.9	5.0	5.4	4.9	4.4	4.4	5.0	5.0	5.0	5.0	5.4	5.1	4.9	4.7	5.0	4.8
**Asn**	4.1	4.8	4.9	5.0	4.5	4.7	4.9	5.0	4.5	4.3	4.4	4.9	4.4	4.7	4.6	5.2	4.7	4.3	4.6	4.7
**Gln**	4.5	5.2	5.0	4.9	4.7	5.1	5.3	4.8	4.9	4.6	4.5	4.9	5.1	4.7	4.7	4.8	5.0	4.6	4.7	5.2
**Trp**	3.5	5.0	4.9	5.4	5.1	4.9	4.9	5.4	4.7	5.1	5.0	4.6	5.1	4.7	5.3	4.9	4.8	4.7	5.2	5.0

A proper estimation of the gap distance 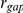 is crucial in the ADC model. The optimal 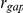 is expected to cover the most intrinsic contact distance between two residues, especially the major preferred contact distance. Meanwhile, the optimal 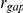 must be small enough to exclude the fake “preferred contact distance” (the linearly increased peak position in **Figure 6**). The appropriate 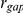 values are estimated statistically from the optimal cutoff distance 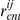. First, 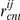 are selected from the valley positions under different gap-distance conditions (**Figure 5**). The valley position does not shift drastically when the gap distance 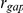 increases. This stable behavior aids us to identify a statistically optimal 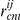. Then, from among all gap distances, there should be one critical value at which all contact-pair distances are less than the optimal 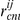. The critical value is the appropriate gap distance 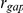.

Take Val–Phe for example, **Figure 5** shows the valley position near 5 Å, i.e. the optimal cutoff distance 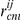 = 5 Å. When the 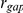 varies from 0 to 8 Å, the positions of occurrence bin extend from 4 to 12 Å along the horizontal axis. At the critical gap-distance value 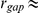
1 Å, all bin positions are lower than 5 Å. In such cases, the optimal gap distance is 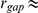
1 Å. Although small fluctuations do occur, we note that the optimal gap distance for all 210 residue pair types is around 1 Å.

Instead of the fixed value of 5 Å, the optimal cutoff distances in **Table 1** provide pair-specific cutoff distances. The ADC model uses the cutoff distance 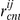, which depends on the specific atom pairs between two side chains (see Equation (9). For the same type of residue pair, such as Val–Phe, the minimal side chain distance may occur between different pairs of atoms and hence the cutoff contact distances are usually different. The maximal and minimal cutoff-distance variations can be seen in **Figure 5**. No matter what cutoff distances are used, either the popular 5-Å criterion or the optimal ones in **Table 1**, single-value cutoff distances can hardly deal with various atom-to-atom contact cases. The 5 Å may be a good choice for two atoms surrounded by hydrogen atoms. However, it may be too large for two atoms that have no hydrogen atoms attached to them. A fixed, large cutoff-distance value is more convenient for most residue side chain contact, but over-estimation can happen when the cutoff distance is adopted for heavy atom pairs in more compactly packed side chains.

### Conclusion

The influence of residue side chain anisotropy has been studied for three side chain contact models. The atom distance criteria (ADC) contact model shows high accuracy in the determination of residue contact and overlaps. Protein structures can be classified as closely packed and loosely packed groups with the help of two different linear fit of 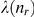 by ADC method. The isotropic sphere side chain (ISS) model has systematically misjudgements in determining of both residue contact and overlaps. The residue surface distances are underestimated and more side chain overlaps emerged. With the different radii in three principle directions, anisotropic ellipsoid side chain (AES) model is more accurate than ISS in determining residue contact. The number of misjudgement is less than 5% of that in ISS method. However, AES need much more computations than ISS model. Based on the algorithm accuracy and complexity analysis, ADC model is recommended as the best all-atom side chain contact determination method. And AES is the most promising coarse-grained method.

## Methods

### Atom distance criteria (ADC) for residue side chain contact and overlap

Two atoms can be considered in ‘contact’ when they are in close interaction. Van der Waals interaction, a commonly employed close interaction, decreases rapidly with the distance between atoms. Residue contact can be defined when any two non-hydrogen side chain atoms (NHSA) from two residues are in the range of van der Waals interaction. This interaction-based contact definition are usually converted to distance-based contact definition by a cutoff distance of van der Waals interaction [56], [57], [58]. A popular cutoff distance between atoms from different residues is 5 Å [97]. We discuss the atom distance criteria in details in this section.

X-ray crystallography can barely resolve hydrogen atoms in most protein crystals. As a consequence, hydrogen atoms are absent in most PDB files. Thus the influence of hydrogen atoms that are attached at the NHSA has to be approximately included in determining residue-contact relations. we define the contact radius 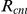 as in the following (**Figure 2** (C)).
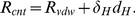
(7)Where 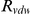 is the van der Waal's radius of the side chain atom; 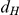 denotes the additional volume thickness if this atom has an attached hydrogen atom; 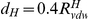 was used in the current study; 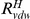 denotes the van der Waals radius of hydrogen atom; and 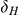 is a constant value , which is defined as:
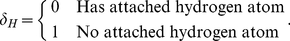
(8)Atom interactions are confined to a limited range, such as the contact radius of an atom. If the distance 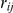 between atom 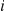 and 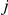 satisfies the criterion 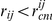, the atoms are considered to be in contact. Other than a predetermined fixed value, the atom-contact cutoff distance 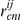 is calculated based on side chain atom-surface distance, which reflects the anisotropy in side chain orientations.
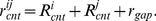
(9)

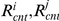
 are contact radii of atoms 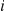 and 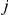. The 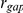 is the gap distance representing the decay of atomic interaction. With the current definition, the cutoff distance 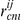 can be different values for different pairs of side chains, or for the same pair of side chain with different torsion angles.

Generally speaking, the atom distance between different residues cannot be less than the sum of covalent radii (the disulfide bond is about 2.05 Å in length, which is almost equal to the sum of covalent radii of the S atom). Overlaps happen when one atom is within the range of the covalent volume of other atoms, i.e., 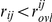.
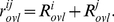
(10)Here 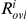 is the ‘overlap radius’ for residue side chain atom 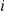.
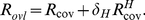
(11)

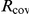
 is the covalent radius of the atom; 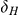 is a constant value as defined in the contact radius; and 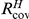 is the covalent radius of the hydrogen atom.

### Isotropic sphere side chain (ISS) contact model

In many coarse-grained protein structure models, the isotropic sphere is used as a simplification of residue side chains [2], [3], [4], [14], [25]. The sphere model depends on two parameters: center position and radius. The geometry or mass center of heavy-atom collections is usually chosen as the sphere-shaped side chain center (**Figure 2** (D)). Although the radius of gyration, 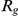, is commonly used in describing the size of the residue side chain, some atoms will be located outside the range of 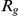. In the present study, the side chain radius is scaled to envelop all atoms. In order to determine the contact and overlap relationships between residues, effective ‘contact radius’ and ‘overlap radius’ have been proposed for sphere-shaped side chains.

The effective ‘contact radius’ 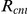 for isotropic sphere side chain is defined as:
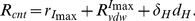
(12)Here 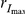 is the maximal radius of all 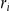. The 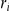 denotes the distance between the side chain atom 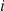 and the center of the sphere. The atom index corresponding to the maximal radius is the 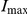. The 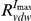 is the van der Waals radius of atom 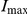; 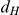 and 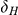 have the same definitions as in the ADC model.

The effective ‘overlap radius’ is defined as:
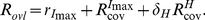
(13)Here 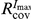 is the covalent radius of atom 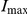. The 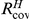 is the covalent radius of the hydrogen atom. Based on the contact and overlap radii of isotropic sphere side chain model, two cutoff distances are proposed:
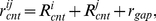
(14)

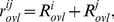
(15)

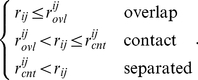
(16)Where 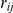 is the distance between the center of the sphere of residue 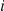 and 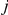; 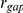 is a gap distance between spherical surfaces, which can be adjusted to provide some flexibility to residue-attraction interactions.

### Anisotropic ellipsoid side chain (AES) contact model

Although the ISS model works well for equi-axial, spheroidal atom systems, the radius-of-gyration techniques do not retain three-dimensional anisotropic properties with regard to side chain orientations. A more general ellipsoid side chain model is proposed in this work. The residue side chain is simulated as ellipsoids with three principal axes for arbitrarily shaped atom clusters. The orientations of resulting ellipsoids are then used to study relative positions of the residue side chain. All residue NHSAs are used to calculate three ellipsoidal radii.

The principal radii of the best-fit ellipsoid are along the transformed Cartesian coordinates axes. Principal axes can be obtained from the diagonalization of matrix 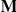.
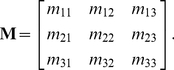
(17)Here, 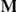 represents the moment of inertia. The elements 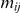 are calculated from the atom positions 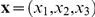 relative to the side chain center 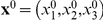, averaged over side chain atom number 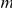 [98], [99]. The subscripts 1, 2 and 3 are coordinate indices.
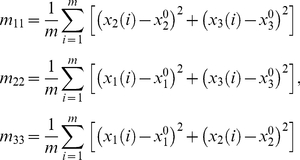
(18)

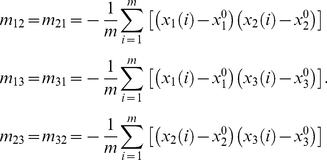
(19)Where 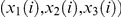 are the coordinates of atom 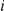.The major and minor radii (known as the principal radius [99]) are determined directly from the Eigen values 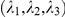 of 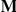.
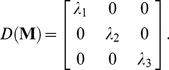
(20)Here 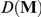 denotes the diagonalization of 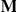 based on the cyclic Jacobi method [100]; 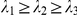 are three eigen values; and 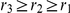 are the major and two minor semi-axes of the best-fit ellipsoid, respectively. If the atomic mass is assumed to be uniform and the side chain to have a unit mass, the eigen values are the principal moments of inertia for the ellipsoid side chain models 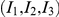.
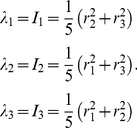
(21)The principal radii of ellipsoid side chain are [99]:
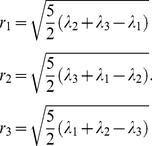
(22)The ellipsoid orientation vector 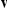, with respect to the reference coordinate can also be obtained from the eigen vectors [98]. Using the ellipsoid side chain model, many spurious side chain overlaps can be avoided (see Results).

Some studies have been reported with regard to the detection of ellipsoid overlap [94], [95]. In this study, we apply the algorithm to residue side chain contact determinations. For two ellipsoids A: 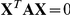 and B: 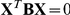, the solution of characteristic equation 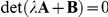 is used as a simple algebraic condition for the separation of the ellipsoids. Here 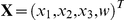, where 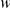 is the 4^th^ dimension that represents the constant term in the ellipsoid formula; 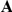 and 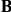 are 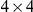 real, symmetric matrices. The interiors of two ellipsoids are represented by 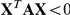 and 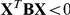. Then,

1. A and B are separated if and only if 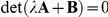 has two distinct positive roots.
2. A and B touch externally if and only if 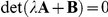 has a positive double root.
3. A and B overlap if their characteristic equation has no positive root.

Matrix 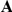 and 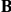 can be constructed with the ellipsoid principal direction vectors 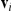 (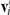 for 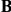) and principal axis radii 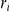 (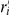 for 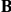) [94].
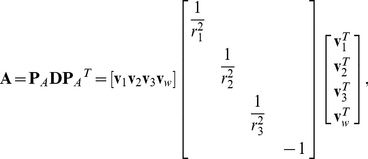
(23)

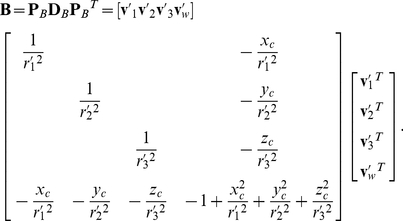
(24)Here 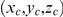 denotes the central position of ellipsoid B relative to the center of ellipsoid A. The fourth dimension of 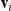 corresponds to the constant term as in vector 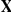.

To avoid unnecessary calculations, two screening conditions are introduced prior to collision detection. The ellipsoid overlap and contact will be evaluated only if the side chain distances 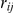 fall within a suitable range.
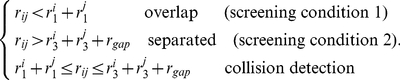
(25)Here 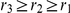 are the major and two minor semi-axes of the best-fit ellipsoid; 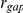 is a gap distance between ellipsoid surfaces, which represents the decay zone of attraction interaction.

If the residue side chain distances clear the first two screening conditions, there still are three possibilities: overlap, contact or separated. According to the ellipsoid collision conditions, overlap can easily be sorted out. The problem is with regard to how contact from separated cases can be discriminated. In a similar manner as in the ADC and ISS models, ‘contact radius’ are proposed for the ellipsoid side chain model. With this contact radius, the ellipsoids can be scaled to include all atoms represented by van der Waals radius. Then, the ellipsoid collision conditions are checked for the enlarged ellipsoids. If the scaled ellipsoids are still separated, the two residue side chains are separated. Otherwise, the residue pair is said to be in contact.

### Analysis of algorithm complexity

For a protein chain with 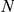 amino acids, the number of NHSA of residue 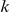 is 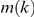. The ADC side chain contact algorithm needs to calculate the distances between two heavy atoms 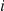 and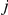. Let 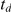 be the complexity of distance operation 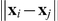. The total complexity of ADC for a whole protein chain is:
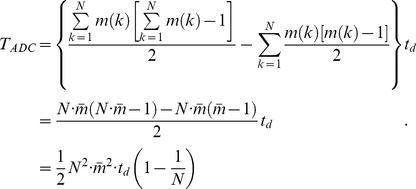
(26)Here 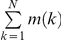 is the total number of NHSA. Distance calculation is not essential for atoms within the same residue. Thus, there is a deduction 
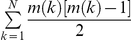
; 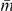 is the average NHSA number in side chain 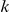 with respect to all the 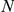 residues. As protein size 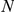 increases to a large value, 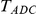 asymptotically approaches 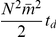.

In the case of the ISS model, there are three main steps. The bulky spherical centers have to be estimated first. Then the sphere radii are determined. Finally, the distance between two side chains is calculated and checked. If the geometrical center is considered the side chain center 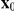, the coordinate-averaging operation 
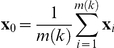
 will be involved in calculations for residue 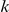. Here 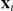 is the location of the non-hydrogen atom 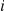. The largest atom-to-center distance 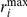 in the side chain 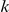 is utilized as isotropic sphere radius. In order to determine the 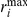, distance operation 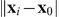 is carried out for all 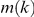 atoms. Finally, the distances between any two side chain centers are calculated and checked.

If 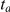 is the approximate complexity of each add operation for 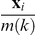, then 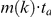 is the complexity of the coordinate-averaging operation 
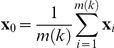
. Let 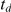 be the complexity of the distance operation 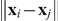, and the total complexity of the ISS model will be:
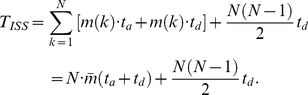
(27)Although residues have different rotamers, the side chain radius will not change too much for such conformational isomers. To simplify the process, the same type of residues is assumed to have the same radius. As a consequence, the calculation of radii is only necessary for 20 types of amino acid, rather than for all the 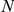 residues. The complexity can be re-written as:
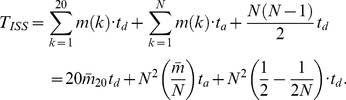
(28)Here 
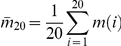
 is the average number of NHSA for 20 amino acids. There is little difference between 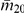 and the average number 
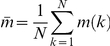
 along the chain. When protein chain length 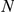 increases to a large value (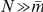), 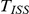 asymptotically approximates to 
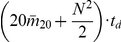
.

The complexity of the AES algorithm is comprised of several aspects: the creation of moment of inertia matrix 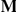, the diagonalization of 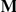 and calculation of principal semi-radii, and the determination of residue contact according to ellipsoid collision conditions.

The elements of 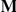 are calculated from the relative positions of side chain atom to side chain center. In the same way as in the ISS algorithm, side chain-center calculation complexity is derived by 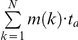. Here 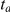 is the approximate complexity related to coordinate-averaging operations. Considering the symmetry of the 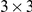 matrix 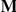, relative position estimations require 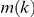 partial distance operations (only two coordinate axes are used) for each matrix element. Let 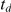 be the complexity of distance operation. Matrix creation has a complexity as:
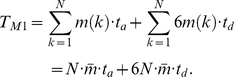
(29)The direct diagonalization of matrix 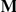 results in an algorithm complexity 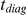, which covers the computing cost of principal radii vectors. The total complexity for the whole protein chain is 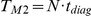.

The ellipsoid-collision conditions are based on the solution of the characteristic equation 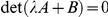. The constructions of 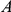 and 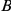 need products of three 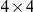 matrices and the complexity is 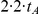. Here 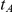 is the matrices multiplication complexity. The number of solutions can be obtained by solving the characteristic equation, which has a complexity independent of protein size and total atom number. This complexity is represented as 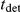. The ellipsoid collision complexity for 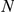 residues is 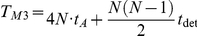.

From all the above analysis, the total complexity of the AES algorithm for an entire protein is:
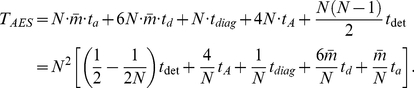
(30)When the protein size 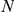 increases to a very large value (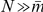), 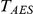 has an asymptotic approximation as 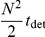.

For large proteins (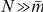), the asymptotic approximation complexity of the ADC, ISS and AES algorithms are 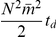, 
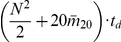
 and 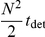, respectively; 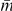 is number of NHSA with respect to all the 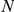 residues in a protein; and 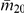 is the average number of NHSA with respect to 20 types of amino acids. The difference between 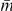 and 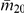 is trivial. Thus, an obvious fact is that the ADC model needs a significantly larger number of computations than the ISS model. The AES appears less complex than the ADC model. However, the 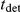 is much larger than 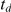. If 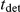 can be written as 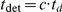, the AES complexity will be 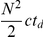. The average number of NHSA usually satisfies 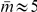. As a consequence, ADC complexity is around 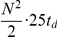. When 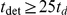, AES complexity exceeds that of the ADC model. In current algorithms, the complexity 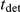 for solving a fourth-order equation 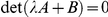 and determining the number of different solutions is significantly greater than 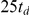. Stated briefly, the AES is currently the most computationally intensive algorithm model.
